# *Arabidopsis* SDG8 Potentiates the Sustainable Transcriptional Induction of the *Pathogenesis-Related* Genes *PR1* and *PR2* During Plant Defense Response

**DOI:** 10.3389/fpls.2020.00277

**Published:** 2020-03-11

**Authors:** Xue Zhang, Rozenn Ménard, Ying Li, Gloria M. Coruzzi, Thierry Heitz, Wen-Hui Shen, Alexandre Berr

**Affiliations:** ^1^Institut de Biologie Moléculaire des Plantes du CNRS, Université de Strasbourg, Strasbourg, France; ^2^Department of Horticulture and Landscape Architecture, Purdue University, West Lafayette, IN, United States; ^3^Center for Plant Biology, Purdue University, West Lafayette, IN, United States; ^4^Department of Biology, Center for Genomics & Systems Biology, New York University, New York, NY, United States

**Keywords:** *Arabidopsis thaliana*, histone, methylation, transcription, RNA polymerase II, biotic stress, salicylic acid, *Pseudomonas syringae*

## Abstract

Post-translational covalent modifications of histones play important roles in modulating chromatin structure and are involved in the control of multiple developmental processes in plants. Here we provide insight into the contribution of the histone lysine methyltransferase SET DOMAIN GROUP 8 (SDG8), implicated in histone H3 lysine 36 trimethylation (H3K36me3), in connection with RNA polymerase II (RNAPII) to enhance *Arabidopsis* immunity. We showed that even if the *sdg8-1* loss-of-function mutant, defective in H3K36 methylation, displayed a higher sensitivity to different strains of the bacterial pathogen *Pseudomonas syringae*, effector-triggered immunity (ETI) still operated, but less efficiently than in the wild-type (WT) plants. In *sdg8-1*, the level of the plant defense hormone salicylic acid (SA) was abnormally high under resting conditions and was accumulated similarly to WT at the early stage of pathogen infection but quickly dropped down at later stages. Concomitantly, the transcription of several defense-related genes along the SA signaling pathway was inefficiently induced in the mutant. Remarkably, albeit the defense genes *PATHOGENESIS-RELATED1* (*PR1*) and *PR2* have retained responsiveness to exogenous SA, their inductions fade more rapidly in *sdg8-1* than in WT. At chromatin, while global levels of histone methylations were found to be stable, local increases of H3K4 and H3K36 methylations as well as RNAPII loading were observed at some defense genes following SA-treatments in WT. In *sdg8-1*, the H3K36me3 increase was largely attenuated and also the increases of H3K4me3 and RNAPII were frequently compromised. Lastly, we demonstrated that SDG8 could physically interact with the RNAPII C-terminal Domain, providing a possible link between RNAPII loading and H3K36me3 deposition. Collectively, our results indicate that SDG8, through its histone methyltransferase activity and its physical coupling with RNAPII, participates in the strong transcriptional induction of some defense-related genes, in particular *PR1* and *PR2*, to potentiate sustainable immunity during plant defense response to bacterial pathogen.

## Introduction

Inside the eukaryotic nucleus, genomic DNA is wrapped around histone octamers [2x(H2A, H2B, H3, and H4)] to form nucleosomes, the basic building blocks of chromatin (Kornberg, 1974). Besides being structurally important to enable DNA to fit into the nucleus, chromatin represents an inherent barrier to all processes requiring access to DNA. Thus, mechanisms such as transcription rely notably on dynamic changes in histone/DNA and/or histone/histone contacts inside chromatin to access target DNA. Dynamic chromatin changes are achieved through different mechanisms and are categorized into different states, ranged from transcriptionally active to poised or constitutively silenced chromatin (Strahl and Allis, 2000).

Protruding from the globular nucleosome core, histone tails may undergo diverse reversible covalent modifications that can either modify the local electrostatic behavior (e.g., acetylation) and/or act as specific docking sites for effectors named “readers” (e.g., methylation; Rothbart and Strahl, 2014). Distinct modifications can act sequentially or in a combined way to bring about specific outcomes, thus constituting a code that considerably extends the information potential of the genetic code (Jenuwein and Allis, 2001). Among the modifications found in higher plants, histone H3 methylation can occur at various lysine residues through the activity of specific enzymes, called histone lysine methyltransferases (HKMTs). In *Arabidopsis*, more than 40 genes encoding putative HKMTs have been classified according to their sequence homology and domain organization into several groups with different lysine specificity (Springer et al., 2003). Among them, members of the Trithorax Group (TrxG), known to catalyze H3K4 and/or H3K36 methylation play pivotal roles in promoting RNA polymerase II (RNAPII) transcription and were demonstrated to control key phase transitions and important stages related to plant development (for a review, see Berr et al., 2011, 2016; Fletcher, 2017). In addition, some TrxG members are also involved in plant defense against pathogens (for review see Bobadilla and Berr, 2016; Ramirez-Prado et al., 2018).

As sessile organisms, plants are challenged by many pathogens in nature. Beside the preformed physical barriers such as cuticle and cell wall as well as constitutive antimicrobial compounds, plant immunity relies on two layers of defense. Firstly, surface membrane-anchored receptors named pattern-recognition receptors (PRRs) can perceive conserved pathogen structures called pathogen-associated molecular patterns (PAMPs), thereby activating PAMP-triggered immunity (PTI). To inhibit/interfere with PTI, pathogens secrete effectors into host cells which in turn can be recognized as avirulence (Avr) proteins by cellular plant receptors inducing effector-triggered immunity (ETI), resulting in much stronger defense responses. In contrast to PTI, ETI is accompanied by a rapid and local programmed cell death at the infection site termed the hypersensitive response (HR) and by the activation of systemic acquired resistance (SAR) in distal tissues of the host (for review see Lee et al., 2017). Downstream in PTI and ETI signaling, the crucial role of phytohormone biosynthesis, signaling pathways and interplays are also well-recognized in effective plant defense against pathogens (Glazebrook, 2005). Salicylic acid (SA) accumulation and signaling are typically associated with plant defense against biotrophs/hemibiotrophs while jasmonate (JA) and ethylene (ET) pathways defend plants against necrotrophs (Pieterse et al., 2012).

Four HKMTs, exclusively from the TrxG group were so far reported to directly or indirectly contribute to the regulation of plant immunity, with their corresponding mutant being more susceptible to some pathogens (Alvarez-Venegas et al., 2007; Berr et al., 2010; Palma et al., 2010; De-La-Peña et al., 2012; Xia et al., 2013; Lee et al., 2016). Among them, the H3K36 di- and tri-methyltransferase SET DOMAIN GROUP 8 (SDG8) was involved in many biological processes, including the regulation of flowering time, organ growth, ovule and anther development, seed development, carotenoid biosynthesis, brassinosteroid-regulated gene expression, light- and/or carbon-responsive gene expression and RNA processing in response to temperature or nitrate signaling (Soppe et al., 1999; Zhao et al., 2005; Dong et al., 2008; Xu et al., 2008; Cazzonelli et al., 2009b; Grini et al., 2009; Tang et al., 2012; Li et al., 2019). Regarding plant immunity, SDG8-mediated H3K36me3 was reported as being crucial for the transcriptional induction of a subset of JA/ET-inducible genes upon infection by necrotrophic fungi (Berr et al., 2010). In addition, the H3K36 methylation deficient *sdg8* mutant was also found more susceptible to hemibiotrophic pathogens and the SDG8 methyltransferase activity was suggested to be required for ETI through the establishment and/or maintenance of a transcription-permissive chromatin state at two *R*-genes (i.e., *RPM1* and *LAZ5*, a RPS4-like R-protein encoding gene; Palma et al., 2010). More recently, *SDG8* together with *SDG25* were proposed to contribute to immunity at least partly through the regulation of *CAROTENOID ISOMERASE2* (*CCR2*) and *ECERIFERUM3* (*CER3*/*WAX2*), two genes encoding enzymes involved in carotenoids and cuticular wax biosynthesis, respectively (Lee et al., 2016).

Salicylic acid (SA) plays an essential role for both local defense and SAR especially against pathogens with a hemibiotrophic lifestyle (Seyfferth and Tsuda, 2014). However, SA has thus far been neglected when addressing the higher susceptibility of *sdg8* to bacterial pathogens. In this study, we provide evidences demonstrating the contribution of SDG8 and H3K36 methylation in connection with RNAPII to the SA immunity pathway in *Arabidopsis thaliana*. We show that despite the higher sensitivity of the *sdg8-1* mutant to different *Pst* strains, ETI still partially operated. We quantified the endogenous level of SA and found that it was abnormally high in *sdg8-1* under normal conditions. During infection, SA accumulation in *sdg8-1* was similar to that in wild-type (WT) at early stage, but then dropped down quickly. In WT, while global histone methylation levels were unchanged upon exogenous SA treatment, local increases of H3K4 and H3K36 methylations and RNAPII loading were detected at several defense-related genes, in particular *PR1* and *PR2*. In agreement with the higher sensitivity of *sdg8-1* to *Pst*, the examined defense-related genes only retained a partial responsiveness to exogenous SA in the mutant and they were compromised in SA-induced elevation of histone methylation and RNAPII loading. In support of a link between RNAPII loading and histone methylation at defense-related genes, we demonstrate that SDG8 can physically interact with the inactive (non-phosphorylated) and active (phosphorylated) forms of RNAPII.

## Materials and Methods

### Plant Material

The *Arabidopsis thaliana* ecotype Colombia (Col0) was used as wild-type (WT) plant. The *sdg8-1* mutant (SALK_065480) in the Col0 background has been previously described (Zhao et al., 2005). The *SDG8:FLAG sdg8* (*EFS:FLAG efs*) was kindly provided by Dr. Yoo-Sun Noh (Seoul National University, South Korea; Ko et al., 2010).

### Pathogen Assays

Pathogen inoculation assays were performed on 5-week-old *Arabidopsis* plants grown on soil in a growth chamber with a 12 h photoperiod and a day/night temperature regime of 22°C/18°C. Bacterial pathogens *Pseudomonas syringae pv. tomato* DC3000 (*Pst* DC3000) harboring an empty vector and *Pst* DC3000 carrying a plasmid-borne *avrRpm1* gene (*Pst avrRpm1*) were used. Bacterial strains were inoculated with a needleless 1 ml syringe as previously described (La Camera et al., 2005) and bacterial growth was determined by counting colony forming units (cfu) as previously described (Katagiri et al., 2002). For RNA extraction, at least six leaves from 10 individual plants were harvested at 0, 1, and 3 days post-inoculation (dpi), pooled and flash-frozen in liquid nitrogen until use.

### SA Quantification and Treatments

For analysis of free SA, inoculated leaves were harvested at 0, 1, 2, and 3 dpi and free SA measurement was performed by ultra-performance liquid chromatography coupled to tandem mass spectrometry (UPLC-MS) on methanolic extracts as previously described using gentisic acid as an internal standard and a 137 > 93 mass transition in negative mode (Aubert et al., 2015).

Salicylic acid treatment was performed by spraying an aqueous solution of 1 mM SA (S5922, Sigma-Aldrich) onto 10-day-old *Arabidopsis* plantlets grown on soil under mid-day length conditions (12 h light/12 h dark) in a growth chamber. Before (0 h), 8, 24 and 48 h after treatment, plantlets were quickly dried and flash-frozen in liquid nitrogen and stored until RNA extraction. For chromatin immunoprecipitation (ChIP), plantlets before and 24 h after treatment were directly fixed in formaldehyde before chromatin extraction.

### Gene Expression Analyses

Total RNA was extracted using the Nucleospin RNA Plant kit (Macherey-Nagel). First strand cDNA was synthesized using Oligo-dT primer and SuperScript^®^ III Reverse Transcriptase (Invitrogen) according to manufacturer’s instructions. The relative transcript abundance was determined in triplicates using gene-specific primers listed in Supplementary Table S1 on a LightCycler 480 instrument (Roche) in a final volume of 10 μL of SYBR Green Master mix (Roche). At4g26410 (*EXPRESSED PROTEIN*, *EXP*) At1g13440 (*GLYCERALDEHYDE 3-PHOSPHATE DEHYDROGENASE*, *GAPDH*) and At4g34270 (*TIP41*) were selected as internal reference genes based on their stability under our experimental condition using geNorm (Vandesompele et al., 2002) and Norm Finder (Andersen et al., 2004). Relative expression values were calculated using the comparative cycle threshold method 2^–Δ^ ^Δ^ ^*Ct*^.

### Western Blotting and Chromatin Immunoprecipitation

Western-blot analysis was performed on histones extracts prepared from 1-week-old seedlings as described previously (Xu et al., 2008). Protein were separated by 15% SDS-PAGE and transferred onto Immobilon-P membranes (Millipore) using a Trans-Blot semi-dry transfer cell (Bio-Rad). Intensity of individual bands was quantified using ImageJ densitometry software (NIH).

Chromatin immunoprecipitation (ChIP) assays were performed according to the previously described method (Liu et al., 2016). Antibodies used to precipitate chromatin were anti-H3 (05-499; Millipore), anti-trimethyl-H3K4 (07-473; Millipore), anti-trimethyl-H3K36 (ab9050; Abcam) and anti-total RNA polymerase II (RNAPII) CTD repeat antibody (ab817, Abcam), together with protein A magnetic beads (Magna-ChIP, Millipore). DNA was purified with the NucleoSpin Gel and PCR Clean-up kit (Macherey−Nagel, Düren, Germany) and analyzed by real-time PCR (LightCycler 480II; Roche in conjunction with the SYBR Green Master mix) using gene-specific primers listed in Supplementary Table S1. Data were analyzed as described in Zhao et al., 2019 for H3K4me3 and H3K36me3 and in Yang et al., 2016 for RNAPII. A mock control was done using uncoupled magnetic beads (Supplementary Figure S5).

### Coimmunoprecipitation Assays

Coimmunoprecipitation assays of SDG8 and the RNAPII proteins were performed using the *Arabidopsis SDG8:FLAG sdg8* transgenic line (Ko et al., 2010). Total proteins were extracted as previously described (Yang et al., 2016). In brief, 10 g of 10-day-old seedlings grown on 1/2 MS-agar plate was grinded in liquid nitrogen and thawed in lysis buffer: 100 mM Tris-HCl, pH 8, 150 mM of NaCl, 2 mM EDTA pH 8, 1 mM MgCl_2_, 1% Nonidet P-40 (74385, Sigma-Aldrich) and 1 mM AEBSF (A8456, Sigma-Aldrich) supplemented with complete^TM^, EDTA-free Protease Inhibitor Cocktail (5056489001, Roche). Lysates were incubated with 0.5 μL/mL of the non-specific endonuclease Benzonase (E1014, Sigma-Aldrich) to degrade DNA and RNA during 1 h at 4°C on a rotating wheel. After incubation, homogenates were cleared by centrifugation. Immunoprecipitation was performed on supernatants using the μMACS DYKDDDDK (FLAG) isolation kit according to the manufacturer’s instructions (130-101-591, Miltenyi Biotec). Proteins bound to magnetic beads were resolved by electrophoresis on 6% SDS-polyacrylamide gel and detected using either monoclonal antibodies against the FLAG tag (F3165, Sigma-Aldrich), against total RNAPII CTD repeat (ab817; Abcam) or against the phosphorylated serine 5 (Ser5P) or Ser2P forms of the RNAPII CTD repeat (C15200007 and C15200005, respectively; Diagenode), followed by a goat anti-mouse-HRP-conjugated antibody (G-21040, Invitrogen).

### Yeast Two-Hybrid Assays

The pGBD-CTD-Kin28, pGBD-CTD-mKin28, and pGBD-Kin28 were kindly provided by Dr. Hisashi Koiwa (Guo et al., 2004). In order to use this tethered system in our yeast two-hybrid system, inserts were amplified by PCR and subcloned into the Gateway donor vector pDONR207 (Stratagene). The SDG8 cDNA was also amplified by PCR and cloned in pDONR207. Next, inserts were fused to the GAL4 binding domain or activation domain using the destination Gateway-compatible pGBT9 (Ghent plasmids collection) or pGADT7 vectors (Clontech), respectively. Plasmids were co-transformed pairwise into the yeast strain AH109 (Clontech) according to the Clontech small-scale LiAc yeast transformation procedure.

The phosphorylation of the CTD was analyzed by western blot using the pGADT7 recombinant constructs containing a hemagglutinin (HA) epitope tag. Indeed, pGBT9 contains a truncated 410 bp ADH1 promoter leading to low expression level making fusion protein hardly detectable by western blot (Van Criekinge and Beyaert, 1999). Protein extracts from yeasts were prepared using the Urea/SDS method described in the Yeast Protocols Handbook (Clontech). Proteins were separated by SDS-polyacrylamide gel electrophoresis (15% SDS-PAGE) and transferred onto Immobilon-P membranes (Millipore). Monoclonal antibodies against the HA tag (H9658; Sigma-Aldrich), against RNAPII CTD (ab817; Abcam) or against Ser5P or Ser2P CTD (C15200007 and C15200005, respectively; Diagenode) were used followed by a goat anti-mouse-HRP-conjugated antibody (G-21040, Invitrogen).

Weak and strong interactions were assayed on synthetic complete medium lacking Leu, Trp and His and containing 25 mM 3-amino-1,2,4-triazole (SD-LWH + 3AT) and lacking Leu, Trp, Ade, His (SD-LWAH), allowing growth for 4 days at 30°C. The synthetic complete medium lacking Leu and Trp (SD-LW) was used as control.

### Statistical Methods

Statistical analyses were performed in R^1^ using a Student’s *t*-test with Benjamini–Hochberg FDR correction.

## Results

### *SDG8* Mutation Increases Susceptibility to *Pseudomonas* Infection

**FIGURE 1:**
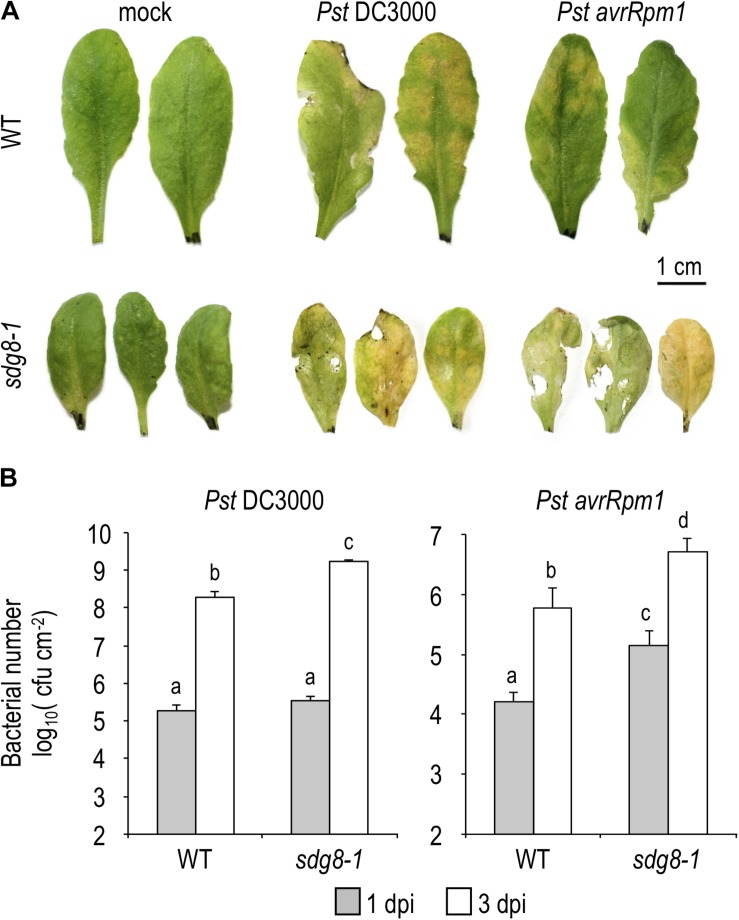
Pathogen-responsive phenotypes in WT and *sdg8-1* mutant plants upon *Pseudomonas syringae* infection. **(A)** Representative leaves of 5-week-old *Arabidopsis* WT and *sdg8-1* mutant plants showing disease symptoms after infiltration with *Pst* DC3000 or *Pst avrRpm1* at 5.10^5^ colony-forming units (cfu/ml). The control treatment (mock) was inoculated with 10 mM MgCl_2_. Photographs were taken at 3 days post-inoculation (dpi). **(B)** Growth of virulent *Pst* DC3000 (left) or avirulent *Pst* DC3000 expressing *avrRpm1* (right) at 1 and 3 dpi in WT and *sdg8-1* leaves of 5-week-old plants. Log transformed data shown are means ± SD (*n* = 12). The experiments were repeated twice with similar results. Letters indicate significant differences (Student’s *t*-test with Benjamini–Hochberg FDR correction, *P* < 0.05).

Hemibiotrophic pathogens such as the bacterial pathogen *Pseudomonas syringae pv. tomato* DC3000 (*Pst*) are known to stimulate SA accumulation and to induce the expression of SA-related defense genes (Seyfferth and Tsuda, 2014). Using the *Pseudomonas*–*Arabidopsis* pathosystem, we investigated the impact of the *sdg8* mutation on the SA-related immunity. Firstly, the susceptibility of the *sdg8-1* mutant to *Pst* was evaluated by syringe-inoculated WT and mutant plants with *Pst* expressing or not the effector gene *avrRpm1* (*Pst avrRpm1* or *Pst* DC3000, respectively). At 3 days post-inoculation (dpi) with *Pst avrRpm1*, *sdg8-1* mutants exhibited more severe symptoms than WT plants, with characteristic tissue necrosis and chlorosis (Figure 1A). In addition, *sdg8-1* displayed significantly higher avirulent bacterial multiplication compared to WT (Figure 1B). We also detected in *sdg8-1* an increased susceptibility and bacterial growth at 3 dpi when the virulent strain *Pst* DC3000 was used (Figures 1A,B). Previously, similar observations were made when the allelic mutant lines *sdg8-2* (Palma et al., 2010; Lee et al., 2016) and *sdg8-4* (De-La-Peña et al., 2012) were infected with *Pst*. In addition, we noted that *sdg8-1* supported higher growth of the virulent than the avirulent *Pst* strain (Figure 1B), suggesting that ETI still operate in the mutant, but less efficiently than in WT plants. Together, our results indicate that in addition to ETI, *SDG8* also regulates PTI/basal resistance in *Arabidopsis*.

### Accumulation of Salicylic Acid Is Compromised in *sdg8-1* Upon Infection

**FIGURE 2:**
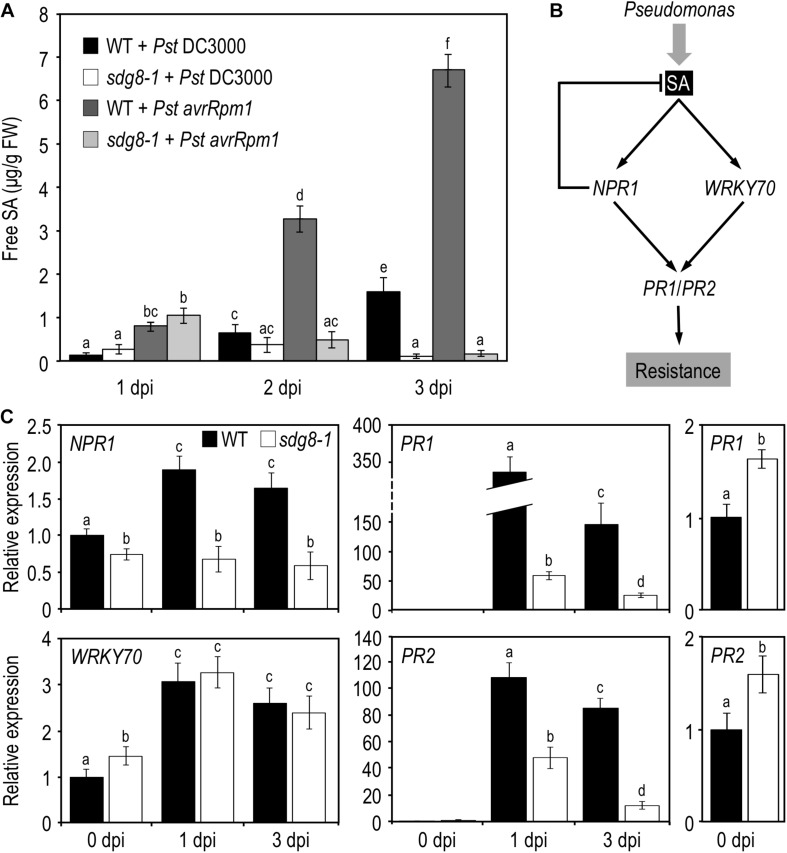
SA accumulation and expression levels of SA pathway-associated genes in WT and *sdg8-1* mutant plants upon *Pseudomonas syringae* infection. **(A)** Quantification of free SA concentrations at the indicated time points in WT and *sdg8-1* leaves of 5-week-old plants after *Pst* DC3000 or *Pst avrRpm1* inoculation. The experiments were repeated twice with similar results. **(B)** Simplified model for the SA signaling network in *Arabidopsis thaliana* depicting genes (in *italic*) analyzed in the present study. **(C)** Expression levels of SA pathway-associated genes quantified by qRT-PCR in WT (black) and *sdg8-1* (white) mutant 5-week-old plants in response to *Pst* DC3000 inoculation. Expression values for each gene are presented relative to the corresponding WT level at 0 dpi (set as 1) as means ± SD (*n* = 3). Inserts on the right are highlighting differential basal expressions observed between WT and *sdg8-1* for *PR1* and *PR2*. The experiments were repeated twice with similar results. Letters indicate significant differences (Student’s *t*-test with Benjamini–Hochberg FDR correction, *P* < 0.05).

Next, we quantified by UPLC-MS the levels of the bioactive free SA in WT and *sdg8-1* mutant plants before and after inoculation with *Pst* DC3000 and *Pst avrRpm1*. Before pathogen infiltration, the steady-state level of free SA was below the limit of detection in WT, but significantly elevated (118.25 ± 39.6 ng/g FW−^1^) in *sdg8-1*. Upon infection, WT accumulated SA in response to both *Pst* strains, with a progressive increase in the course of infection. As expected from the known difference in defense intensity between PTI and ETI, the level of free SA was lower after inoculation with *Pst* DC3000 than with *Pst avrRpm1* in WT. A lower accumulation of free SA in response to virulent compared to avirulent *Pst* was previously reported and the progressive suppression of the PTI-associated SA accumulation by the virulent strain through the production of the phytotoxin coronatine was proposed as an explanation (Carviel et al., 2014). In *sdg8-1*, the SA accumulation at early stage of infection was similar in magnitude to that of WT (Figure 2A). Like WT, *sdg8-1* accumulated SA more rapidly upon infection with the avirulent strain than with the virulent one, further confirming that defense mechanisms triggered by ETI are partially functional in *sdg8-1*. At a later stage of infection, SA continued to accumulate in WT, while the early SA increase detected in *sdg8-1* upon infection with both *Pst* strains vanished at 3 dpi. Together, these results indicate that *SDG8* participates in the negative control of the basal SA level prior to stimulation and promotes SA production/accumulation upon stimulation.

### *SDG8* Is Involved in the Transcriptional Regulation of Certain SA-Related Genes Before and/or Upon Pathogen Infection

Salicylic acid triggers the induction of a plethora of defense genes and is a major regulator of SAR through a signaling pathway that has been extensively characterized (see for details Janda and Ruelland, 2015 and Figure 2B). The pathogenesis-related (*PR*) defense genes *PR1* and *PR2* encode antimicrobial proteins and are typical markers of the SA-mediated defense system. Their transcriptional induction in response to infection by (hemi)biotrophic pathogens or SA requires the coactivator NON-EXPRESSOR OF PATHOGENESIS-RELATED GENES 1 (NPR1). *NPR1* is constitutively expressed and is weakly responding to exogenous SA treatment (Cao et al., 1998). While NPR1 apparently functions as a transcriptional co-activator, its paralogs NPR3 and NPR4 seem to act as transcriptional co-repressors of *PR* expression (Ding et al., 2018). *SDG8* was previously involved in ETI using avirulent *Pst* strains (Palma et al., 2010; Lee et al., 2016), we hereafter focus on basal resistance against *Pst* DC3000. Because SDG8 likely regulates gene expression, we performed quantitative real-time PCR (qRT-PCR) analyses on uninoculated leaf tissue for the above-mentioned genes. The steady-state mRNA levels of *PR1* and *PR2* were significantly elevated in *sdg8-1* compared to WT (Figure 2C, far right panels). The steady-state mRNA level of *NPR1* was slightly reduced in *sdg8-1*, while *NPR3* and *NPR4* were not significantly affected by the *SDG8* mutation (Figure 2C and Supplementary Figure S1A). Next, we addressed their expression at 1 and 3 dpi with *Pst* DC3000. In agreement with the increased susceptibility of *sdg8-1* to *Pst* DC3000 (Figure 1B), *PR* genes were not efficiently induced in *sdg8-1* compared to WT upon infection *Pst* DC3000 (Figure 2C). More upstream, *NPR1* was not induced at all in *sdg8-1* compared to WT, while *NPR3* and *NPR4* behaved similarly between WT and mutant plants (Figure 2C and Supplementary Figure S1A).

Previously, we demonstrated that *SDG8* plays a crucial role in plant defense against necrotrophic fungal pathogens by regulating a subset of genes within the JA/ET-mediated pathway (Berr et al., 2010). *WRKY70* encodes an important transcription factor regulating cross-talk between SA- and JA-mediated pathways and its expression is induced by SA, in both NPR1-dependent and NPR1-independent manners (Li et al., 2004). Before inoculation, *WRKY70* transcript level was slightly higher in *sdg8-1* compared to WT (Figure 2C), which is in agreement with the higher basal SA level and *PR* genes expression. Highlighting its NPR1-independent transcriptional induction, *WRKY70* was similarly induced in WT and *sdg8-1* upon infection despite the lack of induction of *NPR1*.

Because *sdg8-1* accumulated less SA compared to WT at a later stage of infection, we assessed the expression of genes functioning upstream of SA. Three of them were analyzed by qRT-PCR during infection: *ISOCHORISMATE SYNTHASE1* (*ICS1*, also known as *SID2*) which plays a major role in SA biosynthesis (Garcion et al., 2008; Huang et al., 2010), and, more upstream, *ENHANCED DISEASE SUSCEPTIBILITY1* (*EDS1*) and *PHYTOALEXIN DEFICIENT 4* (*PAD4*) which were reported as being important for plant immunity through both SA-dependent and -independent pathways (Bartsch et al., 2006; Cui et al., 2017). The mRNA level of *ICS1* was higher in *sdg8-1* before infection and only slightly increased compared to the WT control upon infection with *Pst* DC3000. *EDS1* and its co-regulator *PAD4* were found basally downregulated and also less efficiently induced in *sdg8-1* upon infection compared to WT (Supplementary Figure S1B).

**FIGURE 3:**
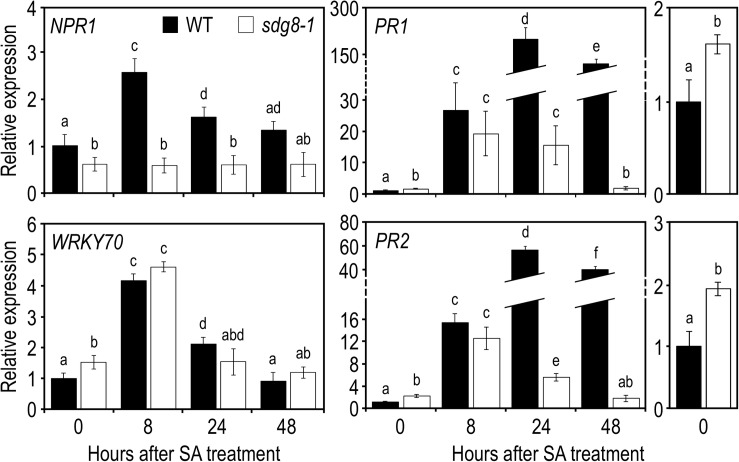
Expression levels of SA pathway-associated genes in WT and *sdg8-1* mutant plants in response to exogenous SA treatment. Expression levels of SA pathway-associated genes in WT (black) and *sdg8-1* (white) mutant 10-day-old seedlings grown in soil and sprayed with 1 mM of SA. Expression values for each gene are presented relative to the WT level at time point 0 (i.e., just before spraying) as means ± SD (*n* = 3). Inserts on the right are highlighting differential basal expressions observed between *WT* and *sdg8-1* for *PR1* and *PR2*. The experiments were repeated twice with similar results. Letters indicate significant differences (Student’s *t*-test with Benjamini–Hochberg FDR correction, *P* < 0.05).

Finally, to determine if the disability of the *sdg8-1* mutant to properly accumulate SA in response to *Pst* DC3000 infection is in part responsible for the weak transcriptional induction of certain SA-related genes, we tested the effects of the exogenous application of SA. SA was applied as a foliar spray on 10-day-old *Arabidopsis* plants, a stage where *sdg8-1* mutant plants are phenotypically indistinguishable from WT plants (Supplementary Figure S2). Upon treatment with SA, *NPR1* stayed uninduced in *sdg8-1* (Figure 3). *WRKY70*, *NPR3* and *NPR4* were all induced by SA in both WT and mutant plants (Figure 3 and Supplementary Figure S3). Interestingly, the mRNA level of *PR1 and PR2* was similar between *sdg8-1* and WT plants 8 h after treatment. Later (i.e., at 24- and 48-h post-treatment), *PR1* and *PR2* expression levels dropped down in *sdg8-1* while they further increased in WT (Figure 3). In summary, *SDG8* appeared to participate in different ways to the regulation of SA-related genes. Before stimulation, *SDG8* controlled the resting-state expression of several SA-related genes. Upon stimulation, *SDG8* seemed to be specifically required for the transcriptional induction of *NPR1*. Finally, at later stages, SDG8 seemed also essential to sustain and further reinforce the induction of the defense genes *PR1* and *PR2*.

### H3K36me3 Deposition by SDG8 Is Required to Widen the Transcriptional Induction of *PR1* and *PR2*

**FIGURE 4:**
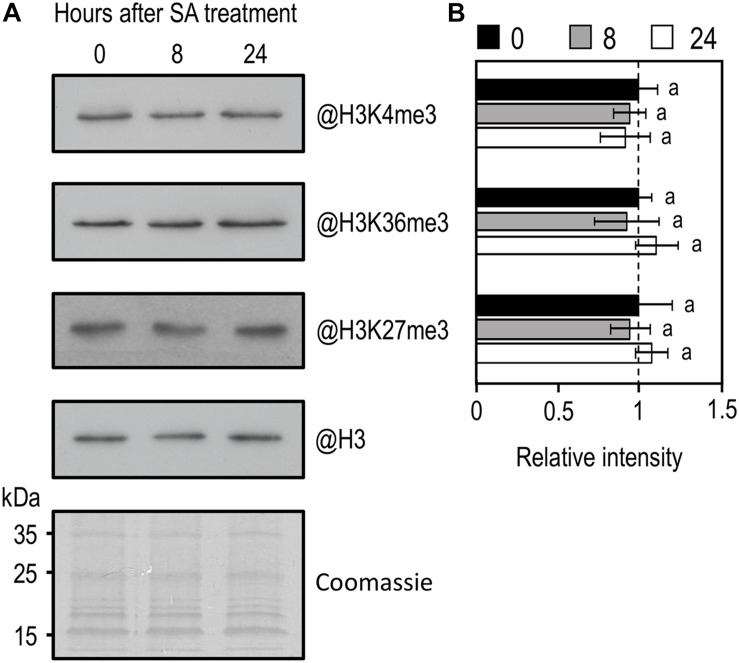
Western-blot analysis of global H3K4me3, H3K36me3, and H3K27me3 in WT plants in response to exogenous SA treatment. **(A)** Amounts of H3K4me3, H3K36me3, and H3K27me3 were determined on nuclear protein enriched fractions extracted from WT 10-day-old seedlings during exogenous SA exposure using indicated antibodies. Histone H3 total protein and coomassie staining were used as a loading control. **(B)** Mean densitometry values ± SEM for H3K4me3, H3K36me3, and H3K27me3 were calculated from at least three independent experiments, normalized to H3 and presented relative to WT. Letters indicate significant differences (Student’s *t*-test with Benjamini–Hochberg FDR correction, *P* < 0.05).

In *Arabidopsis*, like in other eukaryotes, a positive relationship exists between gene expression and the level of active histone marks (i.e., H3K4me3 and H3K36me3), while a negative one exists between gene expression and H3K27me3 (Roudier et al., 2011; Sequeira-Mendes et al., 2014). Because infection of *Arabidopsis* with *Pst* or treatment with exogenous SA triggers massive changes in gene expression (Uknes et al., 1992; Maleck et al., 2000; Schenk et al., 2000), we decided to compare the global level of various histone methylation marks by western blot analysis on nuclear protein extracts from WT plants before and after SA treatment. Interestingly, despite the massive transcriptional changes previously reported, global levels of H3K4me3, H3K36me3, and H3K27me3 remained stable upon treatment with SA (Figures 4A,B).

**FIGURE 5:**
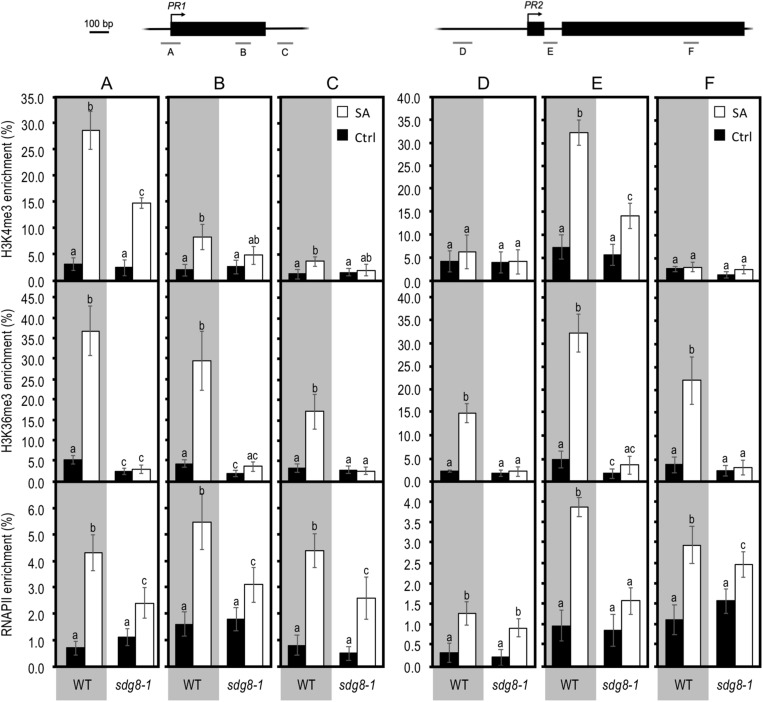
Chromatin immunoprecipitation analyses of H3K4me3, H3K36me3, and total RNAPII at *PR* genes in WT and *sdg8-1* mutant plants in response to exogenous SA treatment. ChIP analyses were used to determine relative levels of H3K4me3, H3K36me3, and RNAPII during treatment with exogenous SA of 10-day-old WT (gray background) and *sdg8-1* mutant (white background) seedlings at the indicated regions of *PR1* (regions A, B, and C) and *PR2* (regions D, E, and F). Genomic structures of the two genes and regions analyzed by ChIP assays are indicated. Black boxes represent exons, arrows indicate TSS and bars labeled from A to F represent regions amplified. The anti-histone H3 was used to normalize H3K4me3 and H3K36me3 levels to nucleosome occupancy. For RNAPII, the DNA enrichment was calculated relative to the input DNA. Mean values ± SD are presented based on results from two biological replicates. Mock controls are provided in Supplementary Figure S5. Letters indicate significant differences (Student’s *t*-test with Benjamini–Hochberg FDR correction, *P* < 0.05).

To further explore the molecular mechanisms underlying the impaired transcriptional induction of certain SA-related genes in *sdg8-1*, we next analyzed the levels of two active histone marks, as well as the amount of RNAPII at some loci using chromatin immunoprecipitation (ChIP). To this end, chromatin was extracted from WT and *sdg8-1* mutant plants before and 24 h after SA treatment. After immunoprecipitation with specific antibodies, DNA was used for quantitative PCR with primers spanning the genic region of several SA-related genes (Figure 5 and Supplementary Figure S4). Firstly, we noticed that basal levels of H3K4me3 and H3K36me3 were weaker for *PR1* and *PR2* compared to *NPR1* and *WRKY70* in WT non-treated plants. Then, the basal levels of H3K36me3 were roughly decreased (with different degree) at *PR1*, *PR2*, *NPR1* and *WRKY70* in *sdg8-1*, whereas the basal levels of H3K4me3 were not significantly changed (Figure 5 and Supplementary Figure S4). This result is consistent with SDG8 being primarily involved in H3K36 methylation (Zhao et al., 2005; Xu et al., 2008; Yang et al., 2014; Li et al., 2015). Finally, the basal levels of RNAPII were largely similar between WT and *sdg8-1* despite the above reported differences in their basal transcription (Figures 3, 5 and Supplementary Figure S4).

**FIGURE 6:**
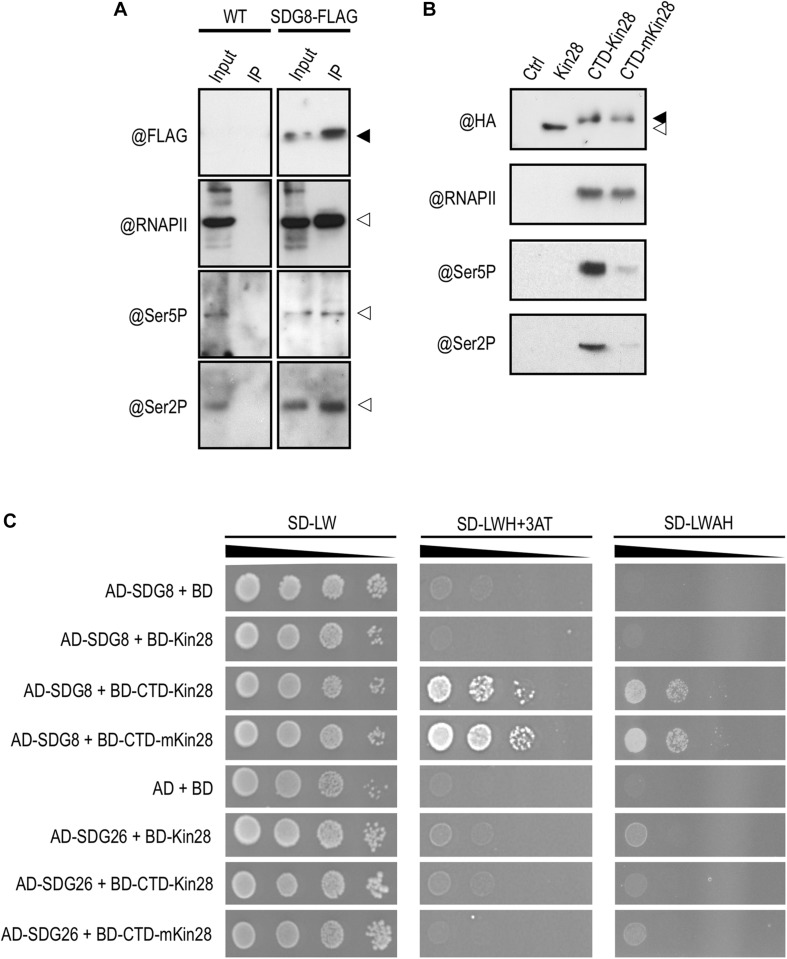
Protein interaction between SDG8 and different phosphorylated forms of RNAPII. **(A)** Proteins were extracted from wild-type (WT) and plants expressing a functional FLAG-tagged SDG8 protein in a *sdg8* mutant background (SDG8-FLAG). An anti-FLAG antibody was used to detect the tagged SDG8 protein (black arrowhead) in the input and after immunoprecipitation using anti-FLAG beads (IP). RNAPII (@RNAPII), the Ser5P form of the CTD of RNAPII (@Ser5P) or the Ser2P form of the CTD of RNAPII (@Ser2P) in input and after IP are indicated by white arrowheads. The experiments were repeated at least twice with similar results. **(B)** The two-hybrid system used to identify protein-protein interactions requiring post-translational modifications was tested for the phosphorylation of the CTD. An anti-HA antibody was used to detect fusion protein in untransformed yeast (Ctrl) and in yeast transformed with the protein kinase Kin28 alone (white arrowhead) or with the CTD fused to either Kin28 (CTD-Kin28) or the inactive Kin28 (CTD-mKin28). Phosphorylation of the CTD detected by antibodies against the Ser5P form of the CTD (@Ser5P) or the Ser2P form of the CTD (@Ser2P) occurs mainly with the tethered wild-type Kin28 but not with the mutated one. The experiments were repeated twice with similar results. **(C)** Yeast two-hybrid analysis of the interaction between SDG8 and the phosphorylated or non-phosphorylated form of the RNAPII CTD. Full-length SDG8 and SDG26 were separately fused to the Gal4 Activation domain (AD), while Kin28, CTD-Kin28 and CTD-mKin28 were fused to the Gal4 DNA Binding domain (BD). Yeast cells co-transformed with the different recombinant vectors were spotted though a series of 10-fold dilutions onto control (SD-LW) and selection media of increasing stringency (SD-LWH + 3-AT and SD-LWAH). The experiments were repeated twice with similar results.

Upon SA stimulation, both H3K4me3 and H3K36me3 levels were strongly increased at the two examined SA-inducible *PR* genes in WT (Figure 5), albeit less pronounced than at *NPR1* and *WRKY70* (Supplementary Figure S4). Concomitantly, RNAPII was also enriched upon treatment at all examined genes, except for *NPR1* where, despite an upward trend, there was almost no statistically significant differences between non-treated and treated WT plants (Figure 5 and Supplementary Figure S4). Compared to *NPR1* and *WRKY70*, the higher enrichment in H3K4me3, H3K36me3, and RNAPII we observed at *PR* genes upon SA treatment might reflect their stronger induction (Figures 3, 5 and Supplementary Figure S4). In *sdg8-1* stimulated plants, the levels of H3K36me3 remained similarly low as before treatment. H3K4me3 was increased in *sdg8-1* after treatment, but to a lesser level than in WT for *PR1*, *PR2* and *NPR1*, or similarly for *WRKY70* (Figure 5 and Supplementary Figure S4). The RNAPII loading was also increased at *PR* genes in *sdg8-1* treated plants (Figure 6). However, further reflecting their non-sustainable transcriptional induction in *sdg8-1* upon SA stimulation, this increase was significantly less pronounced in the mutant compared to WT. Finally, like in WT, the RNAPII loading was largely stable at *NPR1* before and after treatment in *sdg8-1*, while a slight but significant increase was detected at *WRKY70* (Supplementary Figure S4). Combining our q-PCR analyses with our ChIP data let us suppose that the basal and induced trimethylation of H3K36 by SDG8 might be essential to sustain and reinforce *PR1* and *PR2* transcription upon stimulation.

### SDG8 Interacts With Both Phosphorylated and Non-phosphorylated RNAPII CTD

In eukaryotic cells, RNAPII carries out the transcription of all protein-encoding genes into messenger RNAs. Among its various subunits, RNAPII contains a unique carboxyl-terminal domain (CTD) consisting of a large array of heptapeptide repeats in which Serine residues at position two and five are targets of CTD kinases and phosphatases (Dahmus, 1996). Phosphorylation/dephosphorylation events constitute the canonical “phospho-CTD cycle” with (i) unphosphorylated CTD during RNAPII recruitment at promoter, (ii) Ser5P at initiation, (iii) Ser5P and Ser2P during elongation, and (iv) only Ser2P to terminate transcription (Buratowski, 2009). In animal and yeast, SDG8 orthologs were shown to bind preferentially the phospho-CTD of the elongating RNAPII (Kizer et al., 2005; Li et al., 2005). Accordingly, we decided to test this interaction in *Arabidopsis* by co-immunoprecipitation (co-IP) experiments using a rescued *sdg8* mutant line expressing a SDG8-FLAG fusion protein under the control of its own promoter (*efs-*3 EFS-FLAG; Ko et al., 2010). As shown in Figure 6A, SDG8 coprecipitated with RNAPII, as well as with both RNAPII phosphorylated forms. Next, we further explored this interaction using a tethered yeast two-hybrid system in which copies of the CTD were fused to the protein kinase Kin28 (CTD-Kin28), ensuring the CTD phosphorylation, or to the catalytically inactive Kin28 (CTD-mKin28; Guo et al., 2004). We first confirmed by western blot analyses that the Kin28 indeed phosphorylated the fused CTD, while the mKin28 mutated version did not (Figure 6B). Then, we analyzed by yeast two-hybrid the pair-wise interaction of SDG8/CTD-Kin28 and SDG8/CTD-mKin28. An interaction between SDG8 and both the phosphorylated and non-phosphorylated forms of the CTD, but not with the Kin28 alone, was detected (Figure 6C). Because SDG8 and SDG26 are closely related to the yeast sole H3K36-methyltransferase SET2 and to the animal H3K36-methyltransferases ASH1 and HYPB/SETD2 (Xu et al., 2008), we also tested the binding of SDG26 to the CTD and found interaction with neither of the baits (Figure 6C). Together, these results supported an interaction between SDG8 and the inactive (non-phosphorylated) and active (phosphorylated) forms of RNAPII.

## Discussion

In this study, we used the *sdg8-1* mutant (SALK_065480) and found that, like other *sdg8* alleles such as *sdg8-2* (SALK_026442; Palma et al., 2010; Lee et al., 2016) and *sdg8-4* (SALK_014569; De-La-Peña et al., 2012), *sdg8-1* is more susceptible to *Pst* infection (Figure 1). Because the SA-dependent defense pathway has a strong impact on *Pst* growth and plant resistance (Seyfferth and Tsuda, 2014), we explored for the first time the role of *SDG8* in the SA-mediated defense signaling pathway. We quantified SA levels before and during *Pst* infection in WT and *sdg8-1* plants and focused our analyses on several SA-related genes, with a special emphasis on two defense genes, *PR1* and *PR2*. Under non-stressful conditions, we detected in *sdg8-1* an abnormally high level of SA, together with a decreased and increased *NPR1* and *ICS1* basal transcription, respectively (Figures 2, 3 and Supplementary Figure S1). Since *ICS1* plays a major role in SA biosynthesis (Garcion et al., 2008; Huang et al., 2010), its increased basal expression in *sdg8-1* might explain the abnormal accumulation of SA under resting conditions. Given the existence of a negative feedback loop of NPR1 on SA accumulation through the repression of *ICS1* expression (Zhang et al., 2010), our observations suggest that *sdg8-1* has probably lost this retro-control. Because *NPR1* loss-of-function mutants do not constitutively accumulate SA (Genger et al., 2008), SDG8 might also regulate directly or indirectly other genes important to control the basal level of SA.

Even if a functional interplay was reported between SDG8 and a PcG Repressive Complex 2 (PRC2) to repress *Arabidopsis* seed genes (Tang et al., 2012), SDG8 is considered to be primarily involved as a transcriptional inducer (Li et al., 2015). In our study, SA-related genes were falling into three categories based on their basal transcription in *sdg8-1*: downregulated, upregulated and unchanged (Figures 2, 3). Regarding downregulated genes, we detected a decrease in the basal level of H3K36me3 for *NPR1* in *sdg8-1.* As *NPR1* is among the list of SDG8-bounded genes (Supplementary Table S2), these results strongly suggest that SDG8 may act directly to promote *NPR1* basal expression. *NPR1* has five paralogs in *Arabidopsis*, *NPR2*, *NPR3*, *NPR4*, *BLADE ON PETIOLE 1* (*BOP1* and *BOP2*). Interestingly, *NPR3* and *NPR4* were among the lists of SDG8-bounded genes and H3K36me3 hypomethylated genes in *sdg8* (Supplementary Figure S6 and Table S2; Li et al., 2015; Lin et al., 2018). However, in contrast to *NPR1*, their basal expressions in *sdg8-1* were not significantly different from that in WT (Supplementary Figures S1, S3). *WRKY70* was also among these two lists, whereas *PR1* and *PR2* were not. In agreement with this, we found that the basal level of H3K36me3 was reduced for *WRKY70* in *sdg8-1* and only slightly changed for *PR1* and *PR2* (Figure 5 and Supplementary Figure S4). Despite this difference, the basal transcription of these three genes was increased. Because WRKY70 expression is induced by SA (Li et al., 2004), we believe that the higher resting level of SA detected in *sdg8-1* might prevail over the decreased basal level of H3K36me3, thus resulting in an elevated transcriptional steady state. Furthermore, since H3K36me3 was basally low at *PR1* and *PR2* in WT and weakly affected by the mutation of *SDG8*, SDG8 might not be directly involved in regulating *PR1* and *PR2* basal transcription. The higher resting level of SA might, here also, lead to the increased basal expressions of *PR1* and *PR2*. Thus, the relationship between the levels of H3K36me3 and transcription does not seem to be absolute in *Arabidopsis*, and depends on the preponderance of different parameters, such as signaling molecules like SA. Therefore, predicting the transcriptional output from changes in H3K36 methylation seems at certain genes very uncertain and even more complex when considering recent findings involving SDG8 and H3K36 methylation in others gene regulatory mechanisms such as RNA processing and splicing (Pajoro et al., 2017; Li et al., 2019).

When inoculated with *Pst*, *sdg8-1* displayed severe disease symptoms, correlated with enhanced bacterial growth (Figure 1). Despite an accumulation similar to WT at early stage of infection, SA level quickly declined at later stages (Figure 2). Supporting the higher susceptibility of the mutant, we observed that all inductions were substantially lower in *sdg8-1* except for *WRKY70*, *NPR3*, and *NPR4* which behave similarly between *sdg8-1* and WT plants (Supplementary Figure S1). Because WRKY70 is known to induce *PR* genes expression (Li et al., 2004), our result implies the involvement of *SDG8* in inducing the expression of genes at different levels along the SA-dependent signaling pathway. Such a positioning was further confirmed using exogenous SA as a stimulus, since *WRKY70*, *NPR3* and *NPR4* were similarly induced in the mutant as in WT, while *NPR1* upstream of *WRKY70* and *PR* genes downstream were inefficiently induced in *sdg8-1* (Figure 3). NPR1 is known as a central transcriptional regulator critical for transducing the SA signal into activation of most SA-dependent genes (Wang et al., 2005). However, the maintenance of an ETI and the incomplete loss of *PR* genes expression in *npr1* mutants have suggested that certain SA-related responses could be NPR1-independent (Shah et al., 2001; Rairdan and Delaney, 2002). Supporting this idea, a core EDS1/PAD4 pathway working independently of and in parallel with the ICS1-generated SA was recently proposed to maintain important SA-related resistance programs, thereby increasing robustness of the innate immune system (Cui et al., 2017). In addition, the transcriptional induction of *EDS1* and *PAD4* in *Arabidopsis* was previously proposed to be under the control of the Elongator complex subunit 2 (ELP2), likely through modulating the *EDS1* and *PAD4* histone acetylation/methylation status (Wang et al., 2013). Because EDS1 and PAD4 are misregulated in *sdg8* before and upon stress, it would be of interest to further explore the involvement of SDG8 in controlling the EDS1/PAD4 pathway.

Using exogenous SA, we also demonstrated that levels of H3K4me3, H3K36me3, and H3K27me3 were globally unchanged in hormonally treated WT plants (Figure 4). Previously, global changes in histone methylation, especially H3K4me3 and H3K36me3, were observed in WT plants after infection with virulent and avirulent strains of *Pst* (De-La-Peña et al., 2012; Lee et al., 2016). Thus, global changes in some histone marks seem to occur upon *Pst* infection in *Arabidopsis*, while it is not the case using exogenous SA as a stimulus. Even if these observations deserve further analysis especially because of differences in the materials used (i.e., 3 weeks old plants in De-La-Peña et al. (2012), 4 to 5 weeks in Lee and colleagues and 1 week in this work), they suggest the existence of a yet unknown mechanism/effector, probably produced by the bacterial pathogen, enabling *Pst* to modify some histone methylation marks, certainly to promote host susceptibility. Two examples of histone modifying enzyme inhibitors produced by plant pathogens were reported so far, all functioning as potential histone deacetylase (HDACs) inhibitor: (i) the HC-toxin, produced by the maize pathogen *Cochliobolus carbonum* and required for its pathogenicity (Brosch et al., 1995; Ransom and Walton, 1997); and (ii) the depudecin, produced by *Alternaria brassicicola* and required for virulence in *Brassica oleracea* but not in *Arabidopsis* (Cazzonelli et al., 2009a; Wight et al., 2009).

Next, we used exogenous SA treatment to follow chromatin changes specifically at several SA-related genes. We found that both H3K4 and H3K36 methylation were increased upon treatment in WT at all the SA-related genes we analyzed. This finding is in agreement with previous works in which treatments using stress related phytohormones or hormone analogs were reported to provoke changes in histone methylation at some hormone responsive genes in WT (Berr et al., 2010; Jaskiewicz et al., 2011; Kim et al., 2013). Previously, a strong quantitative correlation was described between the level of histone methylation and the level of expression upon stimulation for some developmental transitions (Brusslan et al., 2015; Engelhorn et al., 2017; You et al., 2017). Similarly, *PR1* and *PR2* in our work experienced the strongest H3K4 and H3K36 methylation increase and transcriptional induction upon stimulation in WT (Figure 5). In addition, we found that the level of H3K4me3 was generally increased upon stimulation for all analyzed genes in both WT and *sdg8-1*. Compared to H3K4me3, the level of H3K36me3 was increased in WT and almost unchanged in *sdg8-1* upon SA stimulation (i.e., except at one position of *NPR1* were an increase occurred in *sdg8-1*). Together with the less efficient transcriptional induction of *PR1* and *PR2* detected in *sdg8-1* and the correspondingly lower loading of RNAPII at their chromatin, our results suggest that SDG8 may establish a chromatin context necessary to reinforce and further potentiate the initial induction of strongly SA-responding genes such as *PR* genes. Regarding *NPR1*, the chromatin profiles obtained upon stimulation of WT and mutant plants indicate that SDG8, besides directly regulating the basal expression of *NPR1*, might also regulate its induction. Finally, *WRKY70* was induced similarly between WT and *sdg8-1* upon SA stimulation despite its stably low level of H3K36me3. Previously, *WRKY70* was reported to be basally downregulated and inefficiently induced upon infection in plants mutated for the H3K4 tri-methyltransferase ARABIDOPSIS TRITHORAX1 (ATX1, Alvarez-Venegas et al., 2007). Moreover, H3K4me3 at *WRKY70* nucleosomes was lacking in *atx1*. Together with our results, these data suggest that H3K4me3 might play a more decisive role than H3K36me3 to define the transcriptional outcome of *WRKY70* both basally and upon stimulation.

In animals and yeast, the SDG8 orthologs co-purify with RNAPII and bind preferentially to CTD repeats simultaneously phosphorylated on both Ser2 and Ser5 (Tanny, 2015). This together with the enrichment in H3K36me3 toward the 3′-end of genes spearheaded the link between H3K36 methylation and transcription elongation in animals and yeast (Wang et al., 2009). In *Arabidopsis*, we and others (Yang et al., 2016; Zhong et al., 2019) demonstrate that SDG8, like its orthologs, can bind RNAPII. However, while SDG8 orthologs preferentially bind the phosphorylated CTD of elongating RNAPII, we found that SDG8 can interact with both the inactive (non-phosphorylated) and active (Ser5P and/or Ser2P) forms of RNAPII (Figure 6). In contrast to SDG8, the H3K4 methyltransferase ATX1 was reported to preferentially bind, like its animal and yeast orthologs, the Ser5P CTD of initiating RNAPII (Ding et al., 2011). In addition, while histone methyltransferases classically hold a so-called SET catalytic domain, only SDG8 presents a CW domain (Pontvianne et al., 2010). The CW domain is found in a small number of chromatin-related proteins in animals and plants, and is thought to be a “reader” of methylated H3K4 (Hoppmann et al., 2011). The one found in SDG8 has preference for H3K4me1/me2 (Liu and Huang, 2018). SDG8 thus combines both an H3K4 methylation-reading module and an H3K36me3 writing module, making it the only reader and writer of histone methylation. The presence of this CW domain and the affinity of SDG8 for the phosphorylated and non-phosphorylated forms of CTD might partly explain the distinctive distribution of H3K36me3 in plants compared to animal and yeast, with a peak in the 5′-half of the gene (Roudier et al., 2011). Our data, together with previously published results, indicate that *Arabidopsis* most likely employs SDG8 and H3K36 methylation differently compared to its paradigm usage in animal and yeast. Regarding the impact of a decreased level of H3K36me3 at genes responding strongly to stress (e.g., *PR1* and *PR2*; Figures 3, 5), it is tempting to imagine that in the course of evolution, plants, as sessile organisms, have hijacked the H3K36me3 mark to build up a plant-specific process responsible for the efficient transcriptional induction of genes strongly induced by stresses.

## Data Availability Statement

The datasets generated for this study can be found in the NCBI SRA SRX749018.

## Author Contributions

AB, TH, GC, YL, and W-HS conceived idea, designed research, and wrote the manuscript. XZ, AB, RM, TH, and YL performed experiments and analyzed data.

## Conflict of Interest

The authors declare that the research was conducted in the absence of any commercial or financial relationships that could be construed as a potential conflict of interest.

## Footnotes

1 http://www.r-project.org/

## Supplementary Material

FIGURE S1 Expression levels of SA-related genes in WT and *sdg8-1* mutant plants upon *Pseudomonas syringae* infection. Expression levels of **(A)** *NPR3* and *NPR4*, as well as of **(B)** *PAD4*, *EDS1*, and *ICS1* were quantified by qRT-PCR in WT (black) and *sdg8-1* (white) mutant 5-week-old plants in response to *Pst* DC3000 inoculation. Expression values for each gene are presented relative to the corresponding WT level at 0 dpi (set as 1) as means ± SD (*n* = 3). The experiments were repeated twice with similar results. Letters indicate significant differences (Student’s *t*-test with Benjamini–Hochberg FDR correction, *P* < 0.05).

FIGURE S2 Phenotype of 10-day-old seedlings used for SA treatment. Representative phenotype of 10-day-old wild type (WT) and *sdg8-1* mutant plantlets grown on soil under mid-day length conditions (12 h light/12 h dark) in a growth chamber. Scale bars = 0.5 cm.

FIGURE S3 Expression levels of the *NPR1* paralogs *NPR3* and *NPR4* in WT and *sdg8-1* mutant plants in response to exogenous SA treatment. Expression levels of *NPR3* and *NPR4* in WT (black) and *sdg8-1* (white) mutant 10-day-old seedlings grown in soil and sprayed with 1 mM of SA. Expression values for each gene are presented relative to the WT level at time point 0 (i.e., just before spraying) as means ± SD (*n* = 3). The experiments were repeated twice with similar results. Letters indicate significant differences (Student’s *t*-test with Benjamini–Hochberg FDR correction, *P* < 0.05).

FIGURE S4 Chromatin immunoprecipitation analyses of H3K4me3, H3K36me3, and total RNAPII at *NPR1* and *WRKY70* in WT and *sdg8-1* mutant plants in response to exogenous SA treatment. ChIP analyses were used to determine relative levels of H3K4me3, H3K36me3, and total RNAPII during treatment with exogenous SA of 10-day-old WT (gray background) and *sdg8-1* mutant (white background) seedlings at the indicated regions of *NPR1* (regions G, H, and I) and *WRKY70* (regions J, K, and L). Genomic structures of the two genes and regions analyzed by ChIP assays are indicated. Black boxes represent exons, arrows indicate TSS and bars labeled from G to L represent regions amplified. The anti-histone H3 was used to normalize H3K4me3 and H3K36me3 levels to nucleosome occupancy. For RNAPII, the DNA enrichment was calculated relative to the input DNA. Mean values ± SD are presented based on results from two biological replicates. *ACT2* was used as a control since its transcription is unchanged in *sdg8-1* compare to wild-type and not induced by exogenous SA (data not shown). Letters indicate significant differences (Student’s *t*-test with Benjamini–Hochberg FDR correction, *P* < 0.05).

FIGURE S5 Mock controls of ChIP experiments presented in Figure 5. The anti-histone H3 was used to normalize the mock control of H3K4me3 and H3K36me3. Differences between mock and H3K4me3 or H3K36me3 are indicated (-: *P* > 0.01; ^∗^*P* ≤ 0.01; Student’s *t*-test with Benjamini–Hochberg FDR correction). The mock control for RNAPII was calculated relative to the input DNA. Differences between mock and RNAPII are indicated (-: *P* > 0.01; ^∗^*P* ≤ 0.01; Student’s *t*-test with Benjamini–Hochberg FDR correction).

FIGURE S6 ChIP-Seq results of *NPR1*, *NPR3*, and *NPR4* in *sdg8* and WT plants. Profiles for the distribution of H3K36me3 along the gene-coding region of *NPR1*, *NPR3*, and *NPR4* in *sdg8-5* mutant (red) and WT plants (blue) were obtained from Li et al. (2015). The Y-axis is the RPM normalized ChIP read counts of H3K36me3.

TABLE S1 PCR Primers used to quantify mRNA levels and chromatin modifications.

TABLE S2 List of genes bound by SDG8 among 121 genes known to function in the host response to pathogen challenge in *Arabidopsis*.
