# Supplementary figures and images for: Arabidopsis SDG8 Potentiates the Sustainable Transcriptional Induction of the Pathogenesis-Related Genes PR1 and PR2 During Plant Defense Response
Source: Front Plant Sci. 2020 Mar 11;11:277. doi: 10.3389/fpls.2020.00277 (PMC7078350; doi:10.3389/fpls.2020.00277)

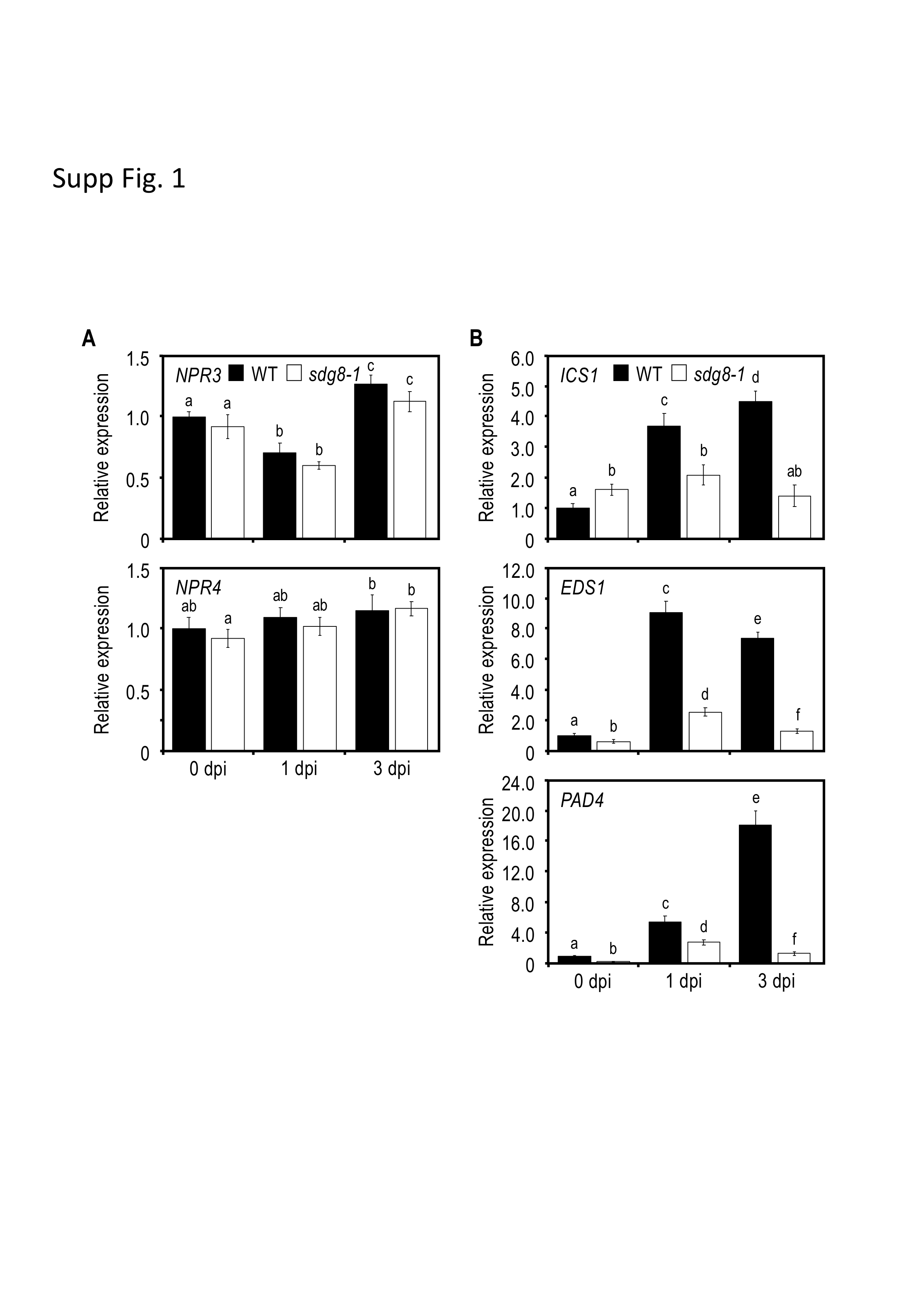

Supplement: FIGURE S1 — Expression levels of SA-related genes in WT and sdg8-1 mutant plants upon Pseudomonas syringae infection. Expression levels of (A) NPR3 and NPR4, as well as of (B) PAD4, EDS1, and ICS1 were quantified by qRT-PCR in WT (black) and sdg8-1 (white) mutant 5-week-old plants in response to Pst DC3000 inoculation. Expression values for each gene are presented relative to the corresponding WT level at 0 dpi (set as 1) as means ± SD (n = 3). The experiments were repeated twice with similar results. Letters indicate significant differences (Student’s t-test with Benjamini–Hochberg FDR correction, P < 0.05). [file Image_1.TIFF]

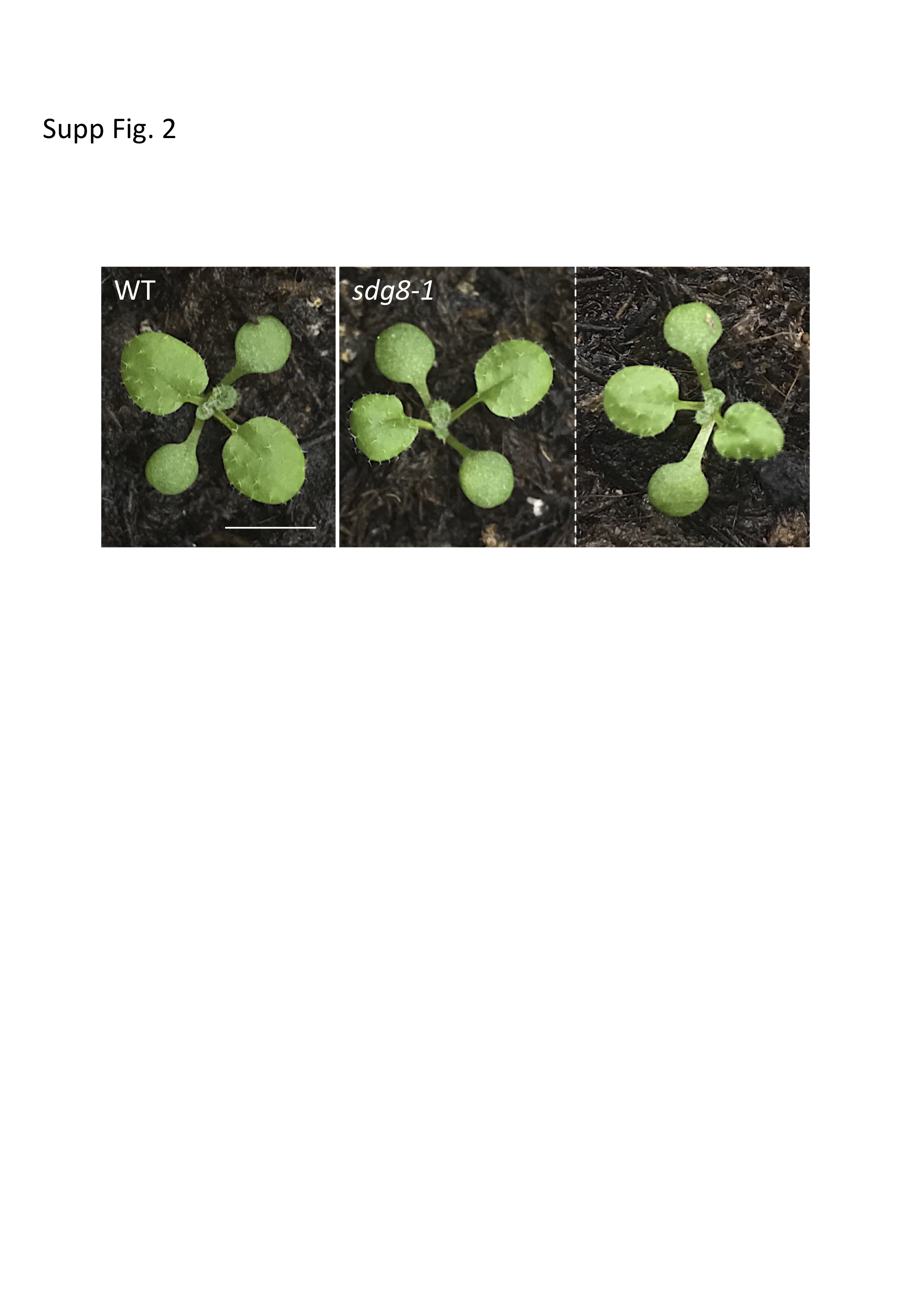

Supplement: FIGURE S2 — Phenotype of 10-day-old seedlings used for SA treatment. Representative phenotype of 10-day-old wild type (WT) and sdg8-1 mutant plantlets grown on soil under mid-day length conditions (12 h light/12 h dark) in a growth chamber. Scale bars = 0.5 cm. [file Image_2.TIFF]

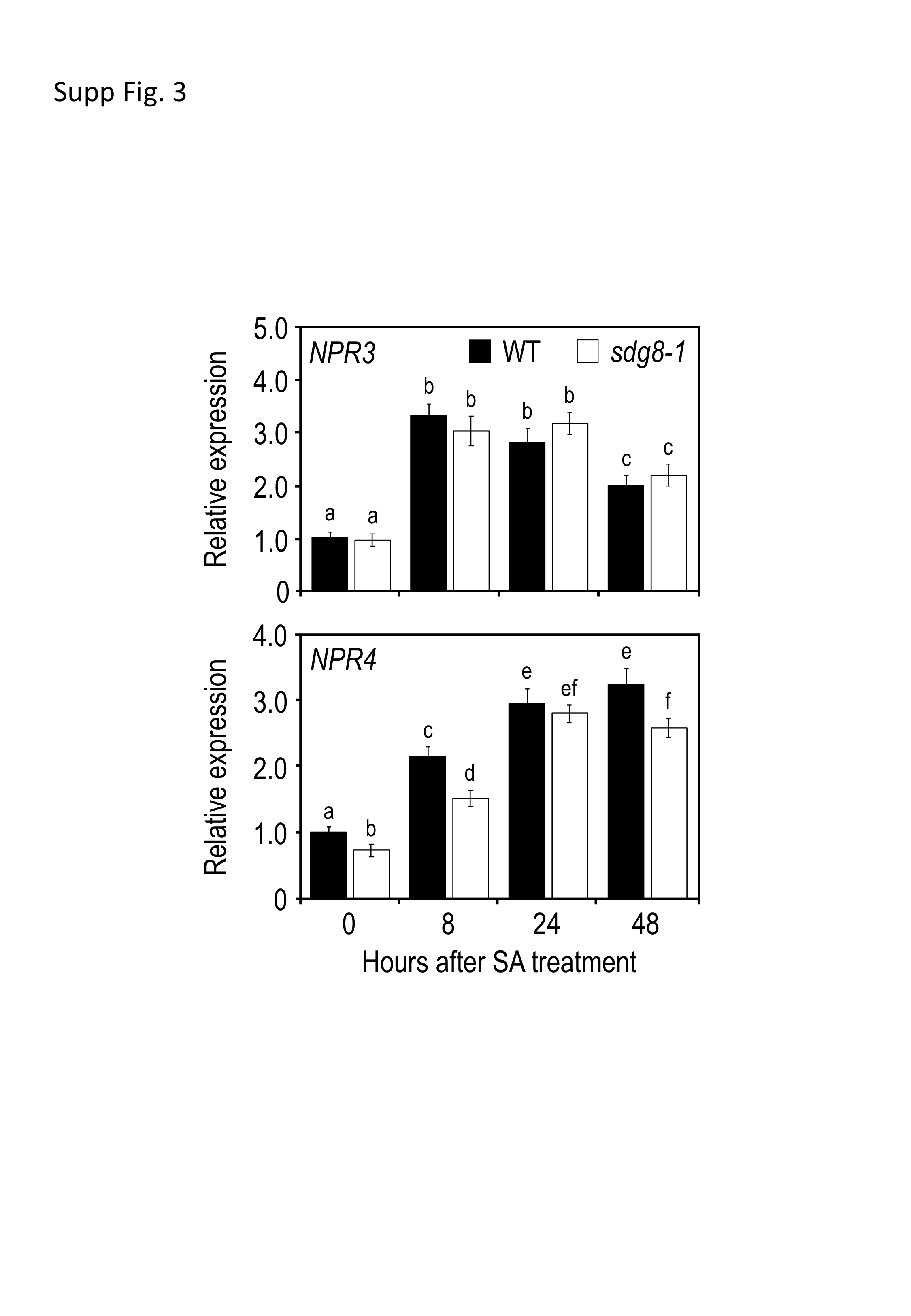

Supplement: FIGURE S3 — Expression levels of the NPR1 paralogs NPR3 and NPR4 in WT and sdg8-1 mutant plants in response to exogenous SA treatment. Expression levels of NPR3 and NPR4 in WT (black) and sdg8-1 (white) mutant 10-day-old seedlings grown in soil and sprayed with 1 mM of SA. Expression values for each gene are presented relative to the WT level at time point 0 (i.e., just before spraying) as means ± SD (n = 3). The experiments were repeated twice with similar results. Letters indicate significant differences (Student’s t-test with Benjamini–Hochberg FDR correction, P < 0.05). [file Image_3.TIFF]

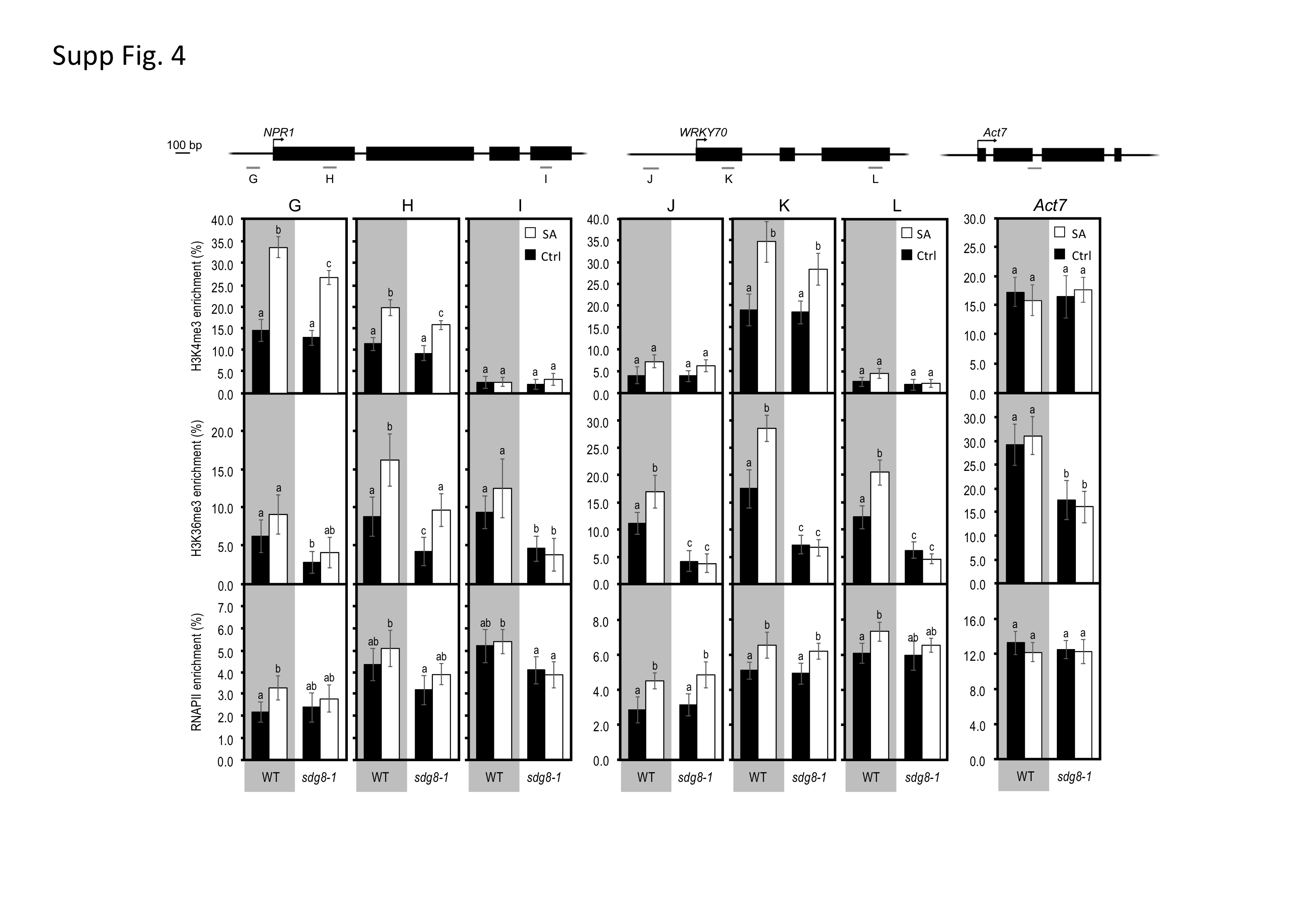

Supplement: FIGURE S4 — Chromatin immunoprecipitation analyses of H3K4me3, H3K36me3, and total RNAPII at NPR1 and WRKY70 in WT and sdg8-1 mutant plants in response to exogenous SA treatment. ChIP analyses were used to determine relative levels of H3K4me3, H3K36me3, and total RNAPII during treatment with exogenous SA of 10-day-old WT (gray background) and sdg8-1 mutant (white background) seedlings at the indicated regions of NPR1 (regions G, H, and I) and WRKY70 (regions J, K, and L). Genomic structures of the two genes and regions analyzed by ChIP assays are indicated. Black boxes represent exons, arrows indicate TSS and bars labeled from G to L represent regions amplified. The anti-histone H3 was used to normalize H3K4me3 and H3K36me3 levels to nucleosome occupancy. For RNAPII, the DNA enrichment was calculated relative to the input DNA. Mean values ± SD are presented based on results from two biological replicates. ACT2 was used as a control since its transcription is unchanged in sdg8-1 compare to wild-type and not induced by exogenous SA (data not shown). Letters indicate significant differences (Student’s t-test with Benjamini–Hochberg FDR correction, P < 0.05). [file Image_4.TIFF]

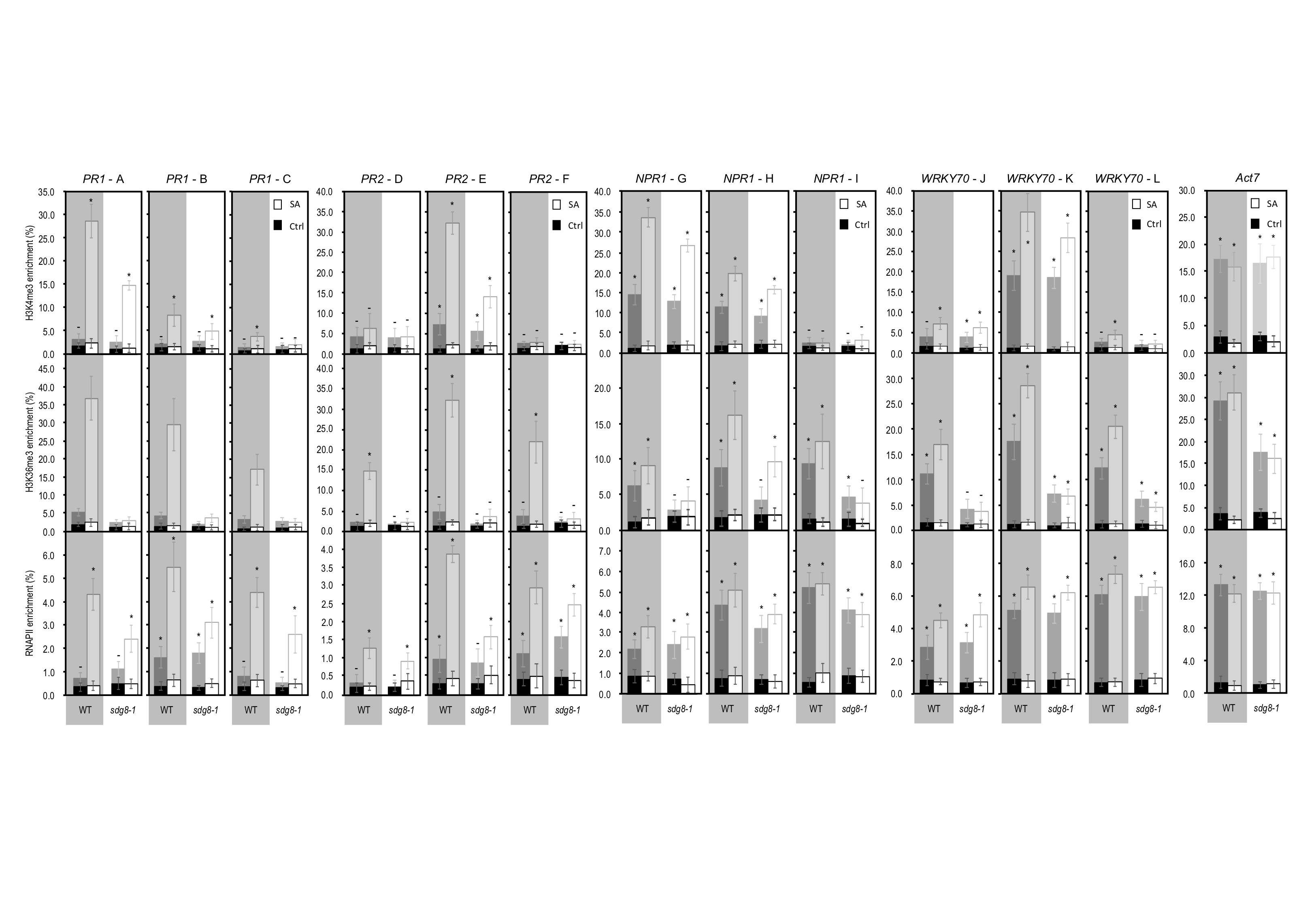

Supplement: FIGURE S5 — Mock controls of ChIP experiments presented in Figure 5. The anti-histone H3 was used to normalize the mock control of H3K4me3 and H3K36me3. Differences between mock and H3K4me3 or H3K36me3 are indicated (-: P > 0.01; ∗P ≤ 0.01; Student’s t-test with Benjamini–Hochberg FDR correction). The mock control for RNAPII was calculated relative to the input DNA. Differences between mock and RNAPII are indicated (-: P > 0.01; ∗P ≤ 0.01; Student’s t-test with Benjamini–Hochberg FDR correction). [file Image_5.TIFF]

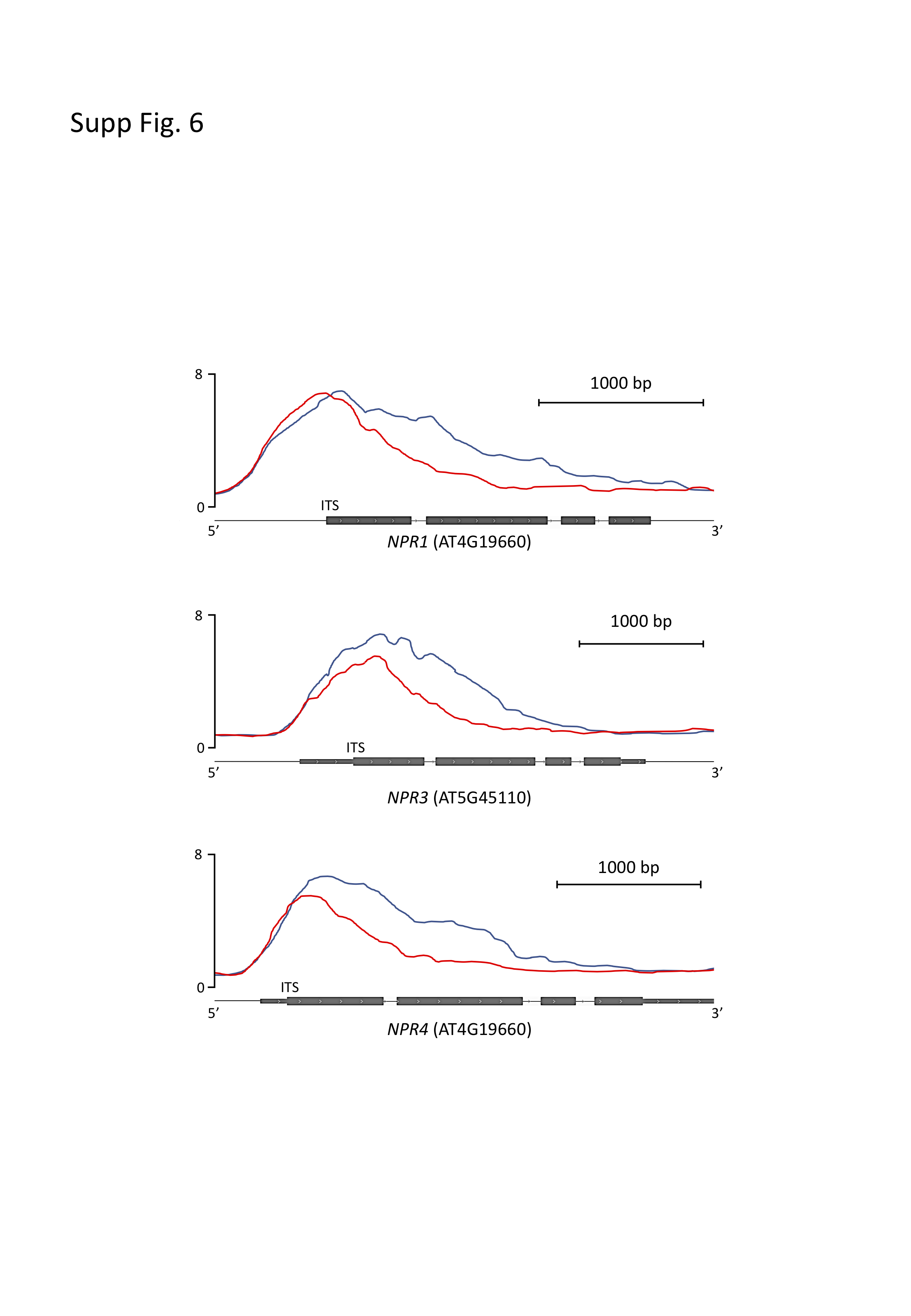

Supplement: FIGURE S6 — ChIP-Seq results of NPR1, NPR3, and NPR4 in sdg8 and WT plants. Profiles for the distribution of H3K36me3 along the gene-coding region of NPR1, NPR3, and NPR4 in sdg8-5 mutant (red) and WT plants (blue) were obtained from Li et al. (2015). The Y-axis is the RPM normalized ChIP read counts of H3K36me3. [file Image_6.TIFF]
